# Alkaloids in Contemporary Drug Discovery to Meet Global Disease Needs

**DOI:** 10.3390/molecules26133800

**Published:** 2021-06-22

**Authors:** Sharna-kay Daley, Geoffrey A. Cordell

**Affiliations:** 1 Natural Products Inc., Evanston, IL 60202, USA; sharnakaydaley@yahoo.com; 2 Department of Pharmaceutics, College of Pharmacy, University of Florida, Gainesville, FL 32610, USA

**Keywords:** alkaloids, drug discovery, fourth industrial revolution, quintuple helix, neglected tropical diseases, multidrug resistance, genomics, artificial intelligence

## Abstract

An overview is presented of the well-established role of alkaloids in drug discovery, the application of more sustainable chemicals, and biological approaches, and the implementation of information systems to address the current challenges faced in meeting global disease needs. The necessity for a new international paradigm for natural product discovery and development for the treatment of multidrug resistant organisms, and rare and neglected tropical diseases in the era of the Fourth Industrial Revolution and the Quintuple Helix is discussed.

## 1. Introduction

### 1.1. Alkaloids, Drug Discovery, and the Fourth Industrial Revolution

Alkaloids are a paradox and an enigma. Inexplicable, contradictory, mysterious, and yet essential for all forms of life. There are the alkaloids we love as spices (capsaicin, piperine, and the *Murraya* alkaloids), those we fear as toxins (batrachotoxin, strychnine, and aconitine), those which affect perceptions in the brain (psilocin, *N*,*N*-dimethyltryptamine, and ibogaine), and there is caffeine, the global stimulant of coffee, tea, maté, and guarana. There are those alkaloids which can relieve pain and, in another context, elicit pain and enormous disruption in society (morphine and cocaine), and alkaloids whose diverse impacts can strain health care systems (nicotine). There is also a plethora of alkaloids from diverse sources which serve as medicines (paclitaxel, vincristine, the cephalosporins, the penicillins, atropine, pilocarpine, quinine, vincamine, etc.). A recent review has provided a concise introduction to plant alkaloids and their broad biological impact on human health [1], and some of the biosynthetic aspects of alkaloids have been summarized [2].

There have been several excellent reports of the role of natural products in drug discovery and the challenges to be faced [3,4,5,6,7,8,9,10,11]. The terrestrial and marine biomes are recognized as essential providers of new source opportunities for natural product discovery [12,13,14,15]. This review will examine the need for new medicinal agents, the evolving strategies for natural product drug discovery, and how the various facets of the Fourth Industrial Revolution (4IR) [16,17], interfacing with the activities embodied in the Quintuple Helix [18,19], provide new opportunities for the development of natural products [20]. The focus will be on the role of alkaloids in the complex milieu of the evolving discovery process, particularly for overcoming multiple drug resistance (MDR), and for the treatment of rare and tropical diseases, including the neglected tropical diseases (NTDs). It will approach the question of how the present and future levels of technological development in the 4IR can enhance the utilization of alkaloids as drugs in new ways to address these global health needs and the welfare of the patient. What are the paradigms in the strategies of the discovery programs that need to change for alkaloids as drugs to be sustainable [21]? 

### 1.2. Global Disease Burden and the Need for New Drugs

In a rational world, the priorities for drug discovery would examine the medicinal agent options, and then mirror the existing and projected disease burdens of humankind; they do not. Most of the world has no say whatsoever in how disease priorities are addressed in terms of dedicated drug discovery. The nature of the communicable and non-communicable diseases of humankind, their changing demographics, the causes of mortality and the forecasting of needs, are all aspects of the Global Burden of Disease (GBD) data analysis, an ongoing project of the World Health Organization, the World Bank, and the Harvard School of Public Health since 1992 [22]. In October 2020, *The Lancet* editorial in a Special Issue dedicated to the 2019 GBD stated: “It’s time for the global health community to change direction” [23]. The conclusion was reached as the present system of addressing the health care needs of the majority population of the world in terms of drug discovery are not being met [24,25,26,27,28,29]. There is an urgent need for natural and synthetic compounds to be available for biological assessment to address these medicinal agent needs. One of the major natural resources to be examined for drug discovery and development are the alkaloids.

## 2. Introduction to Alkaloids

### 2.1. Background and Origins

In 1805, the first alkaloid to be isolated in crude form, morphine, was reported by Sertürner; from opium, it remains an important medicinal agent [30]. A few years thereafter, the important antimalarial agent quinine was obtained [31]. Now it is realized that alkaloids are everywhere in the biome; in addition to plants and various microorganisms, terrestrial isolations have been reported from reptiles, amphibians, insects, mammals, and birds, as well as a diversity of marine sources including sponges, tunicates, corals, and microorganisms [32].

Currently, at least 60 plant-derived alkaloids are approved as drugs in various countries, together with numerous antibiotics and some compounds from marine sources [33]. Their biosynthetic origin is from l-amino acids (tryptophan, ornithine, lysine, phenylalanine, proline, glutamine, histidine, etc.), with attachments from terpenoid biosynthesis, polyketide biosynthesis, and the shikimate pathway producing a wide range of heterocyclic nuclei [32]. In some alkaloids the nitrogen atom is introduced into a preformed nucleus (e.g., terpenoid alkaloids). These pathway relationships and the breadth and depth of the metabolic pool are being actively pursued [34,35,36,37,38,39,40]. As biologically significant metabolites, alkaloids are often highly potent, and in some instances exclusive in their mode of action and application [2]. New alkaloids with a broad range of contemporary biological activities continue to be isolated and characterized. In 2020 alone, the *Journal of Natural Products* reported 316 new alkaloids with 124 metabolites displaying 25 different biological activities.

About twenty years ago, a discussion of alkaloids in drug discovery focused on the chemotaxonomy, biological testing, and future development of plant-derived alkaloids [33]. Two clear outcomes of that analysis related to the structural diversity and biological assessment of alkaloids. The first was that, of the 135,500 plant-derived natural products known at that time (2000), 21,120 alkaloids represented 15.6% of the structures, and 32.5% of the 5750 natural product scaffolds. On the other hand, only 3.3% of the alkaloids had more than five biological tests associated, and 76.4% of alkaloids had not been subjected to even a single bioassay [33]. An analysis of the 60 pure alkaloids used as pharmaceutical agents globally revealed that the average molecular weight (348.9), the number of NH and OH groups (0.97), and average number of O and N atoms (5.55) all fell within the parameters for a “drug-like” molecule [41,42]. In addition, looking at the profile of alkaloid occurrence in higher plants, about 14.2% of higher plants contain alkaloids, although 153 plant families (674 genera) had not been examined for alkaloids, and 50 plant families had three or fewer characterized alkaloids [33]. The twenty most important alkaloid-containing plant families are (alphabetically) the Amaryllidaceae, Annonaceae, Apocynaceae, Asteraceae, Berberidaceae, Boraginaceae, Buxaceae, Celastraceae, Fabaceae, Lauraceae, Liliaceae, Loganiaceae, Menispermaceae, Papaveraceae, Piperaceae, Poaceae, Ranunculaceae, Rubiaceae, Rutaceae, and Solanaceae. From a strategic perspective, these plant families may be regarded as targets, particularly to explore uninvestigated species used medicinally, or they may be strategically avoided for the fear of redundancy in isolation.

The microbial world is even more diverse and underexplored [43]. The number of bacterial species is estimated to be between 10 million and 1 billion [44], and of the soil bacteria only 0.3% are judged to have been cultured [45]; for the marine environment the number examined thus far is thought to be only 10^−5^%. Of the estimated 1.1 million fungi which are considered to exist, around 100 K are named, and of the estimated 140 K mushrooms, about 14,000 are characterized [46]. Thirty-one bacterial phyla of the 61 known remain to be cultured and studied through metabolomics [47]. In the microbial world of fungi and bacteria Streptomycetes and Actinomycetes are rich sources, and beyond these families the opportunities for new compound discovery from natural, potentially sustainable, sources are truly vast.

### 2.2. The Sourcing of Alkaloids

Where, what, and how to source alkaloids are primary questions for any natural product drug discovery program. That source may be a specialized marine or terrestrial location, from a microbe, an extract or compound library based on traditional medicine reports, a specific taxonomic source, or through in silico binding studies of alkaloid libraries at the active sites of specific enzymes or receptors (vide infra). In the past twenty-five years cyanobacteria have become a significant source of new alkaloid metabolites possessing a range of biological activities [48,49].

Generating and maintaining a meaningful number of pure alkaloids for bioassay, even for a modest in vitro screening program, is a significant challenge. Some alkaloids can be purchased, but source collection and re-isolation would be necessary to truly populate library space. Relief may come from concentrates of alkaloid fractions, but even this approach leads to relatively small (1000–2000) sample numbers. Maintaining the stability of alkaloids, particularly basic alkaloids, requires low temperature storage in an inert atmosphere to avoid transformation to their *N*-oxides. For such alkaloid-based drug discovery to be successful, a different strategy is necessary, requiring centralization of analyzed samples, including semi-purified alkaloid concentrates, and a distribution network for biological testing.

Plants are hosts to both fungi and bacteria which have the capacity for independent metabolite production [50,51]. A recent review summarized the isolation or detection of selected alkaloids known to be biologically active from a widely sourced range of endophytic fungi [52]. Of greater interest from a drug discovery perspective was the range of alkaloids, frequently representing completely new scaffolds, characterized from endophytic fungi in the period 2007–2020 [52]. Many of the isolates were examined only for their cytotoxic or antimicrobial activity, providing a discovery opportunity for in silico and possibly subsequent in vitro evaluation.

### 2.3. Alkaloids in Drug Discovery for Tropical and Neglected Diseases

NTD diseases such as African trypanosomiasis, Chagas’ disease, leishmaniasis, schistosomiasis (bilharzia), filariasis, onchocerciasis, trachoma, and leprosy, are conducted by specific parasites, helminths, and bacteria. These NTDs cause major human suffering in at least 149 countries in the world resulting in over 500,000 deaths and affecting about 1.5 billion people, including 900 million children, and have devastating effects on economies, productivity, education, healthcare systems, and nutrition, as well as producing long-term disabilities [53]. They represent the antithesis of a healthy planet/healthy people goal for sustainable development [54]. 

The *Access to Medicine Index 2021* report offers a detailed analysis of the efforts of 20 leading pharmaceutical companies to provide access to critical medicines (mostly NTDs) for low- and middle-income countries who represent 83% of the global population [55]. For the 16 emerging infectious diseases, the 20 major companies had no development pipeline for 10 of them, leaving the majority of those in need without drug resources. A detailed survey of the status of natural products and their derivatives in the drug approval process globally [56] indicated that, between 1981 and 2019, 363 (30%) of the 1205 small molecule new chemical entities that were approved for clinical use were natural products, natural product derivatives, or botanicals. Eighteen compounds were approved for antiparasitic use in the time frame, reflecting 1.5% approvals for drugs globally over 39 years. Nine of these approvals were for natural products or natural product derivatives, six for malaria, two for onchocerciasis, and one for trypanosomiasis, corresponding to 0.25% approvals for NTDs [56].

In January 2021, the World Health Organization introduced a second roadmap for the twenty identified [57] NTDs “*Ending the Neglect to Attain Sustainable Development Goals*” [58]. Surprisingly, in a “gap” assessment, there was no consideration given to the effectiveness and toxicity of the available drugs for NTDs, and no discussion of the need for drug discovery for the 19 infectious diseases. Importantly, except for ivermectin, the drugs being distributed by the donor pharmaceutical company partners are typically the “old-timer” drugs (e.g., melarsoprol, albendazole, and nifurtimox) [53], with their attendant clinical failings [59]. Establishing mechanisms for innovation and the creation of new drugs was not discussed. These mechanisms are much needed given the current situations with NTDs such as bilharzia, lymphatic filariasis (elephantiasis), and leishmaniasis, the details of which are discussed below. 

Bilharzia - Fresh water snails release the cecaria of flat worms of the genus *Schistosoma* (*S. monsonii*, *S. japonicum*, and *S. haematobium*), and are responsible for the disease bilharzia, also known as schistosomiasis, which is second only malaria in terms of morbidity from parasitic diseases [60]. Disease outcomes are not usually detected until overt complications are diagnosed [61,62]. Praziquantel has been the anthelmintic drug of choice for the past 40 years [63,64], although it is not effective against newly developed worms, and cannot prevent re-infection; there are also concerns regarding resistance [65,66,67]. Epiisopilotulorine (**1**) from the leaves of *Pilocarpus microphyllus* Stapf. ex Wardlew (Rutaceae) showed very modest activity in vitro and in vivo [68]. More promising is piplartine (**2**) from *Piper tuberculatum* Jacq. (Piperaceae) among other *Piper* species [69,70]. It showed good activity at 6.3 μM against the egg-laying, was lethal to the worms at 12.6 μM [71], and had a good SI [72]. It also acted synergistically with dermaseptin against *S. mansoni* [73], although bioavailability was modest [74,75,76]. This is an important hit to be pursued given the level of activity and safety, and that it originates from a sustainable source. More targeted, delivery-focused research, aimed at enhancing the pharmacokinetic profile, is warranted.

**Figure 1:**
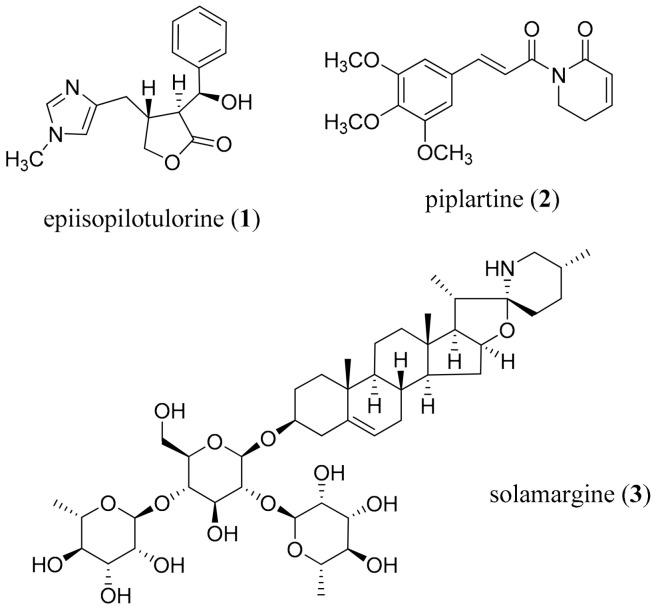
Alkaloids with anthelmintic properties.

Lymphatic filariasis is caused by the filarial worms *Brugia malayi*, *B. timori*, and *Wuchereria bancrofti* [77], and WHO estimates that almost 950 million persons in 54 countries are presently infected, mostly in Africa [53]. Ivermectin is the current drug of choice in combination with diethylcarbamazine, and albendazole, but they are not effective against the adult worm, only the microfilarial stages of the parasite [78]. The most promising alkaloid is the steroidal glycoside solamargine (**3**) from the fruits of *Solanum khasianum* C.B. Clarke (Solanaceae) which was 100% lethal to the worms at 4 mg/mL (Figure 1) [77]. Some efforts to enhance availability through semi-synthesis, and provide analogs to explore the activity profile, have been reported [79].

Leishmaniasis, caused by the *Leishmania* parasite, is transmitted by females of over 90 sandfly species. There are three main forms cutaneous leishmania (CL) caused by *L. major* and *L. tropica* in the old world, and *L. braziliensis* and *L. mexicana* in the Americas [80]; mucotaneous leishmania (MCL) caused by *L. braziliensis* [81]; and visceral leishmaniasis (VL) produced by *L. donovani* in Africa and parts of Asia, and *L. infantum* for infection in North Africa, Europe, and Latin America. Left untreated, VL is 95% lethal, and WHO estimates between 200 K and 400 K cases per year, mostly in Brazil, Ethiopia, India, and the Horn of Africa [53]. There are approximately 1 million cases of CL each year, mostly in Brazil, Algeria, Afghanistan, Iran, Syria, and Colombia [53]. MCL is prevalent in Peru, Brazil, and Bolivia [53]. Pentavalent antimonials, such as pentostam, remain the drug of choice [82], even though severe side effects include the destruction of veins and pancreatitis. Pentostam has been available since the 1940s, and although second-line drugs, such as amphotericin B, miltefosine, paromomycin, and sitamaquine are available, profound side effects are observed, as well as drug resistance. From these few examples, the need to bring a heightened focus to the global NTD burden [83], and to multiple drug resistance (MDR) in infectious diseases, with their tremendous human costs [84,85], clearly remains.

### 2.4. Antibiotic Drug Discovery

The need for new classes of antibiotics for fungal and bacterial pathogens that are pan-resistant to the current drugs is dire and well-documented [56,86,87,88,89,90]. Overall, infectious disease is the second leading killer in the world, and the third in developed nations [91], with 17 million patients per year dying of bacterial infections [92]. The WHO has designated the ESKAPE pathogens (*Enterococcus faecium*, *Staphylococcus aureus*, *Klebsiella pneumoniae*, *Acinetobacter baumannii*, *Pseudomonas aeruginosa*, and *Enterobacter* spp.) [93], which kill over 700 K patients a year, as being of particular concern [94]. The Centers for Disease Control and Prevention in the US have listed 18 drug-resistant pathogens requiring attention in their latest report, of which five are categorized as urgent [95]. Unfortunately, even when small pharmaceutical companies are successful in developing a drug, such as plazomicin (**4**) by Achoagen, the post-approval financial (sales of $80 million) and regulatory challenges may be too large a burden for economic survival. This represents a grave situation for global health care scientifically and financially, which needs to be addressed with great urgency [86]. 

The major issue in antibiotic drug discovery lies in the consistent emergence of antibiotic resistant strains of microbes due to the production of virulent genes encoding protective mechanisms. These include biofilm formation, beta lactamases, etc. Mitigation of the progression of antibiotic resistance includes more controlled use and stewardship of use, while maintaining access in middle- and low-income countries, coupled with improved local diagnostic testing to target more succinctly an effective antibiotic therapy for the individual patient [86]. In addition to controlling more aggressively the use of 30 different antibiotics [96] as animal and plant growth promoters [97], chemically based strategies to overcome drug resistance include: (i) seeking new antibiotic scaffolds through in-field or bioinformatics approaches, (ii) combining antibiotics in therapy, (iii) using adjuvants in combination or alternately for treatment, and (iv) structure modifications of existing antibiotics [43].

One of the classic examples to circumvent ineffective β-lactam therapy is the use of clavulanic acid (**5**) as an inhibitor of β-lactamases for therapy using cephalosporins [98]. Other alkaloids which can conduct these modulations include the phenothiazines [99,100,101]. Berberine (**6**), a well-established antimicrobial agent [102,103,104,105], inhibits biofilm formation of drug-resistant *E. coli* through the downregulation of quorum-sensing related genes [106]. It is available in good yield from several sources, including *Berberis vulgaris* L. (Berberidaceae), *Coptis chinensis* Franch. (Ranunculaceae), and *Hydrastis canadensis* L. (Ranunculaceae). Berberine (**6**) showed an additive effect with ampicillin in vitro against MRSA, and a synergistic effect with oxacillin [107]; activity against inflammatory bowel syndrome (IBD) was recently discussed [108]. The effectiveness of berberine (**6**) in treating diarrhea in adults and children in 38 controlled clinical trials in China [109], prompted synthetic analog development [110]. Berberine (**6**) shows strong inhibitory activity against the sortase of *S. aureus* [111]. Sortases are Gram-positive membrane-bound cysteine transpeptidases, and control multiple virulence mechanisms [112]. It is therefore a sustainably sourced alkaloid, already a commercial entity, and a prime candidate for repurposing. 

**Figure 2:**
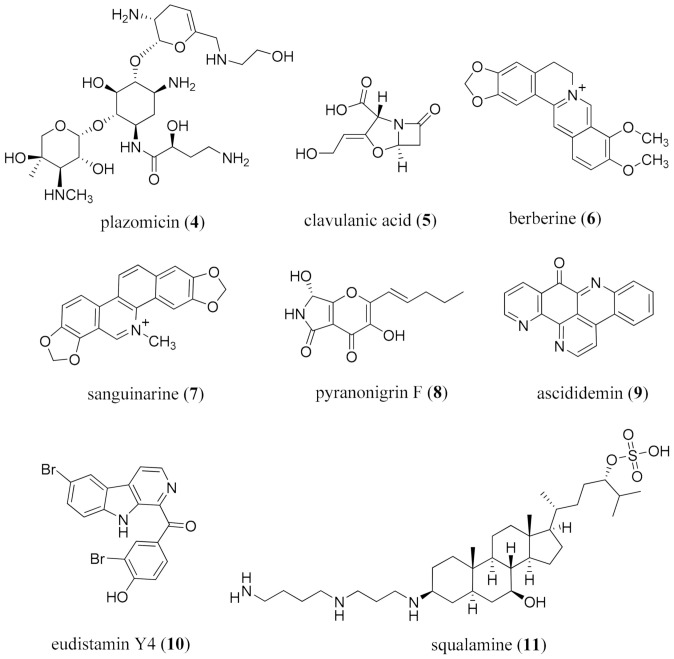
Alkaloids with promising antibacterial properties.

Other alkaloids to be considered include sanguinarine (**7**) and pyranonigrin F (**8**). Sanguinarine (**7**), from *Sanguinaria canadensis* L. (Papaveraceae) and *Macleaya cordata* (Willd.) R.Br. (Papaveraceae), has shown both anticancer [113] and antimicrobial [114] activity, and was formerly used for its antiplaque activity in mouth washes, where it exhibits anti-*Candida* biofilm and anti-gingivitis activity [115,116]. Sanguinarine (**7**) exhibits potent activity against the ichthyotoxic, parasitic ciliate *Ichthyophthirius multifiliis* in carp [117], activity against phytopathogenic fungi [118], and potent effects on *Schistosoma mansoni* in vitro [119]. Pyranonigrin F (**8**) was isolated from the mangrove-associated endophytic fungus *Penicillium brocae* MA-231 [120]. It showed more potent activity than controls against *S. aureus* and the Gram −ve bacteria *Vibrio harveyi* and *V. parahemolyticus*, and antifungal activity against *Alternaria brassicae* and *Colletotrichum gloeosprioides*, Previously isolated antimicrobial alkaloids from marine sources include ascididemin (**9**) from the ascidian *Cystodytes dellechiajei* [121], eudistomin Y4 (**10**) from the ascidian *Synoicum* sp. [122], and the sterol sulfate derivative squalamine (**11**), bearing a spermidine moiety attached at C-3, from the dogfish shark *Squalus acanthias* L. (Squalidae) (Figure 2) [123,124].

### 2.5. Constraints for Alkaloids in Drug Discovery

The absence of established pipelines in Big Pharma for alkaloids to overcome antibiotic drug resistance, and for the development of medicinal agents for rare and neglected tropical diseases, malaria, and tuberculosis, represents a significant time gap in the translation of a compound from in vitro active “hit” to clinical assessment. In addition, there are several significant experimental constraints which impede efforts of alkaloid drug discovery. The inclusion of a selectivity index was mentioned previously; other constraints include declining biological resources, the feasibility of testing, dereplication for known and bioassay-interfering compounds, and residual complexity. 

Regarding declining resources, the diminishing supply of unexplored natural resources, due to deforestation in some of the most biodiverse countries, is not conducive to natural product drug discovery initiatives, particularly when that occurs in low-income areas of the world and in vulnerable populations [125,126,127]. Biodiversity losses through overharvesting in the wild for traditional medicines impact local health care and obviate the discovery of bioactive compounds from those sources before they are examined scientifically; the eons old symbiotic relationship between humans and natural medicine is lost forever [128]. Sustainable supply based on the originating resource evolves as an essential consideration [129,130] and becomes an acute issue when taking a natural product after it is declared a “hit”, and then possibly a “lead”, placing it in line for advanced biological, pharmacological, and toxicological evaluation.

On the feasibility of testing, the failure to test isolates often relates to the physical distance between chemistry and biology; isolation and characterization are in one location, collaborative testing facilities are elsewhere. A second aspect of failure to test is based on availability. Small scale isolation procedures (conserving solvent and chromatographic resources) typically lead to limited quantities for biological assessment in more than one or two assays after structure elucidation, and may abrogate confirmatory tests. In addition, if the isolate is from a complex matrix, it may be challenging to acquire milligram quantities of more than 6–8 metabolites. Molecular networking [131,132] provides a clearer picture of the parent masses present, the structural relationships within a class of potential isolates in the matrix, and their novelty.

Concerning dereplication, the most important aspect of bioactivity-directed fractionation is to avoid the repeated isolation of compounds known to be active in the respective assay. This issue was recognized by Pharma in the early days of antibiotic discovery, and chemical dereplication was a subsequent concern in industry when dealing with plant-based drug discovery [133]. As it minimizes wasted resources and time on dead-end active extracts, dereplication for known bioactives has evolved as an essential element in the early discovery phase of natural product extracts [134] and is greatly facilitated by the access to integrated isolation, bioactivity, and mass spectral datasets (vide infra) [135,136,137,138]. However, it is not sufficient to identify known active compounds in an extract. From a drug discovery perspective, that known active metabolite might not explain the total activity observed in the extract. Consequently, there is an important caveat; namely, it is essential to correlate the biological activity and the UPLC/MS metabolite profile in the same timeframe in order to search by mass for new bioactives directly in the extract through an integrated chemical-biological-database platform [139,140]. 

The global issue for many natural products’ discovery programs, of misusing resources on isolating known bioactive metabolites in an extract, has a new angle; namely, that many widespread compounds respond positively in a broad range of bioassays. These are noted as pan assay interference compounds (PAINS) [141,142] and invalid metabolic panaceas (IMPS) [143]. From an artificial intelligence (AI) perspective, algorithms are needed to identify these compounds (more than one may be present) at an early stage in the analysis of extracts and in extract libraries to avoid their confusion as false positives with truly active metabolites. Fortunately, the alkaloids on the IMPS list are few (berberine, Taxol, tetrandrine, and capsaicin [143]), which may reflect limited distribution based on the taxonomy of other alkaloids and/or limited bioassay assessment. The wide distribution of Taxol in plant endophytes was recently summarized and requires monitoring in plant extracts for its cytotoxic effects [52]. The endophytic metabolites will be present at very low levels, which raises the issue of residual complexity.

Residual complexity becomes apparent when a very minor, undetected, component in a “pure” compound is responsible for the observed, potent activity. Two alkaloids serve as lead examples, sesbanine and sesbanimide [144,145], and rufomyazine and rufomycin [146]. There is also the attempt to trace the anti-TB activity of the highly purified, ubiquitous triterpene ursolic acid [147]. The highest levels of assessed purity still may not detect very low levels of a potent compound, and sometimes only through synthesis or isolation from a different source can that situation become apparent.

## 3. Discovery Strategies

### 3.1. Improved Collaborative Approaches

Extensive collaboration is the key to successful drug discovery programs. To address the absence of a discovery pipeline and the dearth of in-house pharmaceutical company initiatives for long-term and consistent drug discovery for neglected diseases [55], national, regional, and in some instances global leadership is urgently required to respond to the desperate clinical need [86,90]. After the existing partnerships for global initiatives for NTDs was examined [148], the identified health care chasm prompted the development of new collaborative relationships and initiatives [43,148,149,150,151]. Going forward, however, more support from local and regional governments and foundations, as well as venture capital investment for collaborative initiatives, will be essential. One approach is to incentivize innovation for antibiotic drug discovery between small pharmaceutical and biotechnology companies and academia, which would focus on new alkaloid scaffolds and their development [152]. In the present drug approval paradigm, large companies will also be needed to conduct the final clinical development phases under specific licensing agreements. Translational science initiatives [153] to re-examine past drugs “shelved” by pharmaceutical companies is another development pathway for industrial-academic collaboration [154]. In Europe, an Innovative Medicines Initiative has been supported by the EU and by several leading European pharmaceutical companies, including academic clinical investigators [155]. Some of the industry-academic collaborations for drug discovery that have evolved over time to address specific discovery needs, and examined the various approaches, have been described [156]. 

After the poor research record for new NTDs was highlighted [157], the Drugs for Neglected Diseases Initiative (DNDi) was cofounded by the Doctors Without Borders organization using some of their Nobel Peace Prize award [149,158]. Over the years, other global health initiatives have evolved, and one of the challenges has been potentiating the synergy and coherence between these various efforts [159]; a coordinating umbrella organization has been proposed [43]. In 2020, a detailed pipeline analysis of the results from these initiatives revealed 538 candidate drugs for 35 neglected diseases [160]. However, only 68 of these compounds had activity related to the WHO NTDs. It was anticipated that about 600 candidates would be needed for assessment to achieve new clinical entities for each of the 12 NTDs identified, at an overall cost of USD 9.6 billion. The global (43 countries) DND*i* program has afforded six new treatments, 12 new molecular entities, and overseen 25 clinical trials at a cost of about USD 200 million [158]. The explanation for these relatively modest costs relates directly to the practice of “patenting for public health” rather than for exclusivity and profit. These are valuable and innovative programs which need input from diverse sets of natural products, including alkaloids, in the early aspects of discovery. These research initiatives, fostered and supported financially through either the respective government and/or philanthropic foundations, are an important development for all parties involved, will hopefully lead to reducing the gaps in the drug pipeline for rare and NTDs, and find new solutions to the antibiotic resistance crisis. The strong caveat must always be that any new antimicrobial which overcomes resistance is accessible (i.e., available and affordable) to the majority global population. 

### 3.2. Targeted Discovery Based on In Silico Binding

In silico approaches which can selectively identify key compounds, especially alkaloids, without resorting to the massive in vitro screening of huge compound libraries are highly beneficial. They utilize minimal resources, avoid the wasteful time and effort associated with the isolation of pure metabolites, and allow for testing in vitro only candidates with a high probability for activity [129,130,161,162,163]. If an enzyme structure is available for an active site, and has the possibility for the design of inhibitors, then probing with the diversity of known alkaloid structures is a viable strategy to develop potential metabolites for in vitro biological testing. Alternatively, the active site can be assessed through a combination of in silico and in vitro approaches. Although there have been many in silico studies for anticancer compounds and Alzheimer’s derivatives, relatively few studies have examined alkaloids for potential antimicrobial activity, or activity against NTDs or other tropical diseases [164,165,166,167,168]. 

One study sought inhibitors of trypanothione reductase, which is important for the proliferation of Chagas’ disease, and was initiated with twelve structurally diverse alkaloids bearing a conformational semblance to the trypanothione [165]. Of the twelve alkaloids chosen for in silico binding analysis, and considering the desire for sustainable sourcing, steroidal alkaloids from commercial *Solanum* species were selected for further in silico structure development, semi-synthesis, and biological assessment. In another study, in vitro assessment in an inhibition assay with promastigotes of *Leishmania infantum*, *L. braziliensis*, *L. amazonensis*, and *L. guyanensis,* and with intracellular amastigotes of *L. infantum* and *L. amazonensis* indicated that 2,3-dihydro-1-aza-benzo[*de*]anthracen-7-one (**12**) was an inhibitor, which was supported by in vivo activity in mice [166]. Bioinformatics identified four enzymes which could possibly serve in the future as predictors of in vivo antileishmanial activity. A set of 2194 plant-derived metabolites, including 290 diverse alkaloids, were evaluated in silico against three dengue virus proteins, however, the results indicated that polyphenolics, not alkaloids, were the strongest binding compounds [167]. In addition, from an exploratory perspective, in vitro activity of three indole alkaloids from *Rauvolfia tetraphylla* L. (Apocynaceae) against the filarial bovine parasite *Setaria cervi* was correlated with their binding characteristics and in vitro inhibition against glutathione-*S*-transferase (GST) [168]. 

**Figure 3:**
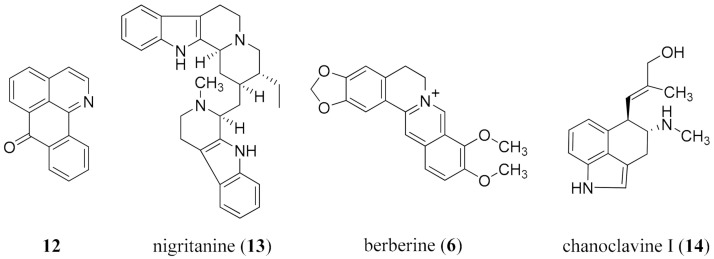
Alkaloids featured in in silico binding studies for antibiotic resistance.

In silico assessment of 39 alkaloids indicated that the β-carboline-monoterpene indole alkaloid nigritanine (**13**) from *Strychnos nigritana* Baker (Loganiaceae) showed high activity against *S. aureus*, which was verified in vitro against three clinical isolates [169]. An in silico model of the extrusor protein MexXY, which causes aminoglycoside resistance of *P. aeruginosa*, was used to explore its inhibition by berberine (**6**) in the presence of tobramycin, and the predicted effectiveness confirmed through in vitro assessment [170]. The ergot alkaloid chanoclavine I (**14**) from *Ipomoea muricata* (L.) Jacq. (Convolvulaceae) had the ability to overcome MDR in *E. coli* and in silico studies confirmed the binding to proteins associated with drug resistance for efflux (Figure 3) [171]. These studies are critically important to establish those enzymes which can best serve as in silico models, thereby functioning as an information source for prioritization to explore the correlations between in silico, in vitro, and in vivo activity.

### 3.3. Alkaloids to Overcome Drug Resistance

**Figure 4:**
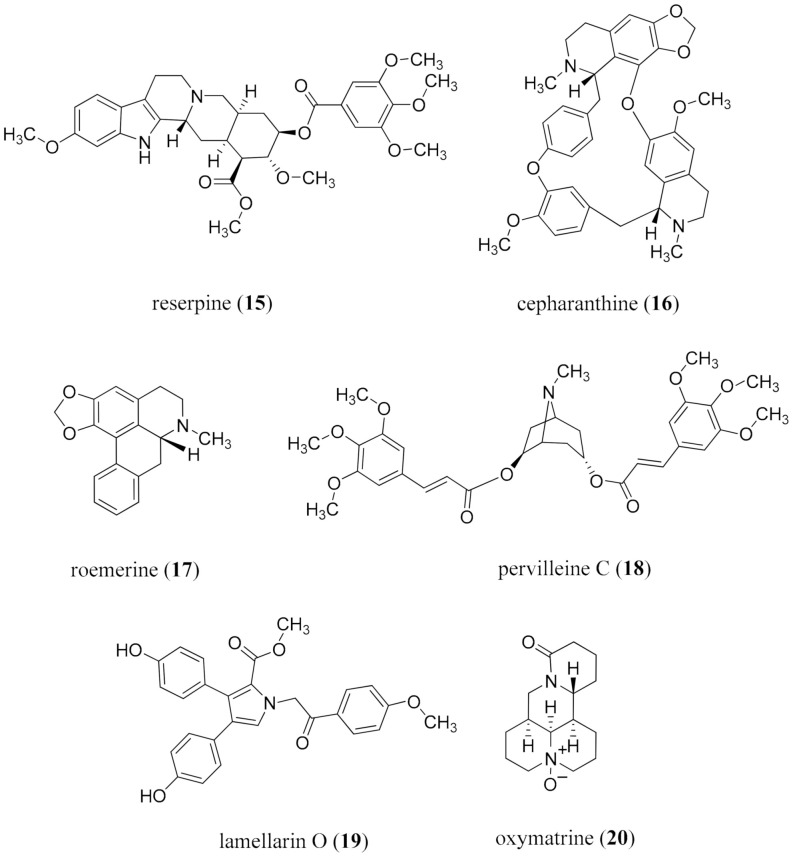
Alkaloids with properties to overcome drug resistance.

In addition to having antimicrobial activity, can alkaloids also serve to overcome the resistance to antibiotics, antimalarial, and anti-TB agents? The monoterpene indole alkaloid reserpine (**15**) is a well-established standard with that capacity [172]. Further searches to find alkaloids to overcome drug-resistance in cancer cells and against drug-resistant malaria indicate that they are rare entities. Some examples include the bisbenzylisoquinoline alkaloid cepharanthine (**16**) [173,174] and related alkaloids [175], aglaiamide derivatives from *Aglaia* species [176], iboga alkaloids from *Peschiera laeta* (Mart.) Miers (Apocynaceae) [177], the aporphine alkaloid roemerine (**17**) from *Annona senegalensis* Pers. (Annonaceae) [178], the tropane polyesters, the pervilleines (e.g., **18**), from *Pervillea* sp. Decne. (Apocynaceae) [179,180], lamellarin O (**19**) from the marine sponge *Ianthella* sp. [181], and oxymatrine (**20**), which overcomes inhibition to hepatitis B infection (Figure 4) [182]. These alkaloids are typically active at a lower dose than either of the two positive control compounds reserpine (**15**) and verapamil. Several of these groups of alkaloids merit further study for their mechanism of action in overcoming drug resistance.

### 3.4. Transformation of Alkaloids

The chemical modification of known alkaloids to explore structure activity relationships (SAR), or to assess for new biological responses, is an important aspect of the drug discovery process, and can be either highly targeted based on lipophilicity (log *P*) or in silico binding considerations. Among the alternative methods to be applied for altering the initial alkaloid structure are chemical transformations, microbial transformations, and biocatalysis. 

Morphine, atropine, cocaine, aconitine, penicillin, cephalosporin, vinblastine, and camptothecin have each served in the past as alkaloid scaffolds for modification to improve potency, diversify their applications, or reduce toxicity [2]. Hundreds of derivatives have been synthesized based on the original alkaloids, and some products, particularly in the morphine alkaloid [183,184,185] and the cephalosporin and penicillin [186,187] series, have become important drugs.

#### 3.4.1. Chemical Transformations

Creating compounds which explore biological space in a rational manner is an important aspect of the discovery process; even though, at least in the past, it may have been an untargeted and somewhat empirical approach, it was the basis for the explorations of combinatorial chemistry with large libraries of synthetic compounds [188]. Modifications of biologically active alkaloids have long been used to explore structure activity relationships and the potentiation of activity or, more recently, to overcome drug resistance to antibiotics. Most of these transformations are straightforward, and in contemporary chemistry some would classify them into the “click” category, particularly when the original sample size is limited [189]. “Click” chemical reactions, which evolved from the combinatorial chemistry strategy for drug discovery, are characterized by consistently high yields, easy to perform, simple clean-up, little or no purification, no need for inert atmosphere or strict exclusion of water, and the use of readily available reagents. Since the first demonstrations, reviews of the strategy from a drug discovery perspective have appeared [190,191,192]. Some of the reaction options include [189]: cycloaddition reactions (1,3-dipolar- and hetero-Diels-Alder); nucleophilic opening of small heterocyclic systems, such as oxiranes and aziridines; formation of ketocarbonyl derivatives; and the products from reactions on double bonds. One can envisage that several alkaloid structure types could benefit from the application of this chemistry to form new compound libraries for assessment.

A quite different approach to developing structural diversity in natural products is to conduct chemical reactions, such as reductions, oxidations, halogenations, aminations, hydrolyses, etc., directly on plant or microbial extracts seeking to modulate the observed biology [128,193]. Beyond the application of acids to serve as hydrolytic reagents, several reports using this approach have resulted in the creation of bioactive compounds from inactive extracts [194,195,196,197], as well as developing new scaffolds for bioactivity assessment [198,199,200]. Chemically profiled extracts of monoterpenoid indole alkaloid-containing plants, whose alkaloids are both highly functionalized and bioactive, would be an interesting and viable target for such an empirical study.

#### 3.4.2. Microbial Transformations

Microbial transformation of purified alkaloids is an alternative, empirical strategy for structure modification, although the yields are typically not high. The original regio- and stereoselective 11α-hydroxylation of progesterone (**21**) by a *Rhizopus* spp. at the Upjohn Co. in 1952 was critical, as it demonstrated the potential to create new centers for chemical development at an unreactive site reproducibly on a large scale [201]. In alkaloid chemistry, it is an under-explored area as a method to introduce functionalization at unreactive sites. In addition, it has not been used extensively for functional group modification or transesterification reactions to explore modifying levels of bioactivity [202,203]. The most important example for an alkaloid is the introduction of a 14β-hydroxy group into the morphinan skeleton by *Pseudomonas putida* M10, which increases analgesic potency, and was studied as a high yielding industrial process for the formation of hydromorphone and hydroxycodone [204,205]. The transformation of nicotine (**22**) by *Arthrobacter nicotinovorans* has also been of industrial interest [206,207]. This neglected area of alkaloid chemistry needs more explorations particularly if yields can be enhanced, and the breadth of organisms studied expanded, with an emphasis on gene manipulation to enhance substrate acceptability. 

**Figure 5:**
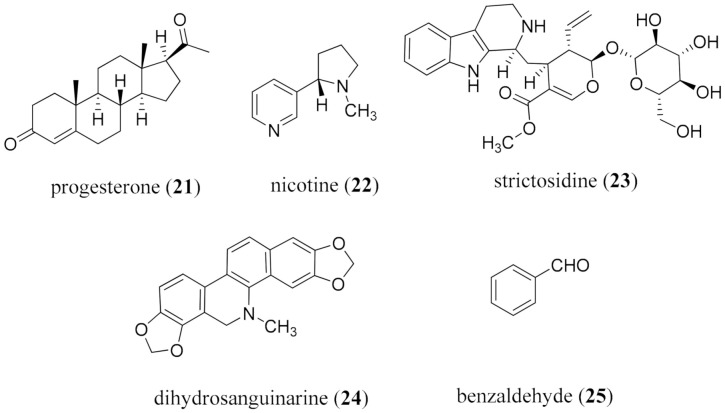
A small selection of alkaloids which have been subjected to microbial transformation, and progesterone (**21**) and benzaldehyde (**25**).

In addition to the functionalization of purified alkaloids, further exploration into microbial transformations as a route to the production of known alkaloids may offer more sustainable approaches to sourcing alkaloids. A good example of this application was demonstrated by Brown et al., where the monoterpene indole alkaloid progenitor strictosidine (**23**), was produced by yeast, and was then modified by the removal of three genes and the introduction of 21 new genes [208]. In 2014, a 10-gene plant pathway in yeast was reconstituted which produced dihydrosanguinarine (**24**) (Figure 5) [209]. Reconstituting plant biosynthetic pathways in microbial hosts as a factory for alkaloid production is proving to be an attractive alternative. In addition to a potentially more sustainable approach to the sourcing of alkaloids, hybrid biosynthetic pathways may also be constructed to facilitate de novo alkaloid modifications [209]. This demonstrates the feasibility of the use of microbial systems as a source of new and known alkaloids [209]. However, further work is required to facilitate the transition to industrial scale drug discovery, some aspects of which have been outlined by Courdevault et al. [210].

#### 3.4.3. Biocatalysis

The use of enzymes for the development of new compounds has a long history [211], beginning with the conversion of benzaldehyde (**25**) with HCN in the presence of emulsin from almonds [212]. Over time, drawbacks in using biocatalysis became evident, including the lack of regio- and stereo-predictability, the low yields, and product feedback inhibition [211]. However, directed evolution involving gene mutagenesis, expression, and functionality screening has approached some of these concerns, and will undoubtedly continue to enhance this as an important approach for alkaloid diversification [213]. More functional details are necessary to foster the precise mutations that can be made for the development of selected, stable, reusable enzymes having wide substrate capacity as robust reagents for oxidations, reductions, esterifications, hydrolyses, and cyclization reactions, etc. [214], and to effect reactions at normally inaccessible sites. 

The combination of enzymatic and non-enzymatic catalysts (cooperative catalysis) offers an alternative to the functionalization of inactivated or inaccessible sites, as well as promoting the interconversion of starting material to enantiomers [215]. There are a few examples of the enzyme engineering [216], one of which includes the use of a bifunctional artificial metalloenzyme comprised of streptavidine engineered with aspartic and glutamic acid residues with a docked biotinylated Rh(III) complex [217]. The metalloenzyme allowed for asymmetric C-H activation, which was used to access dihydroisoquinones [217]. These approaches, however, need more refinements to ensure industrial and sustainable applications. Perhaps a greener and more sustainable process could present itself as a one-pot chemo- and biosynthetic approach [218].

Another facet to consider is that the inclusion of biocatalytic options in AI systems, for consideration in retrosynthetic analysis, is imperative [219] in realizing greener synthetic protocols. The development of bacteria-originating enzymes for specific chemical transformations is also an approach that requires enhanced development, particularly from a commercial perspective to produce catalytic systems which can be stabilized and embedded through 3D-printing and then re-utilized. It is also well-established that whole plant parts can be used to effect high-yielding, enantioselective carbonyl reduction and acyltransferase reactions [220]. 

#### 3.4.4. Application of Nanotechnology

There are multiple scientific reports on the isolation, bioactivity, and bioavailability of alkaloids, each facet with their own drawbacks. Over the years, the emergence and combination of nanotechnology to drug discovery has proven quite beneficial [221]. Reports indicate that the use of nanoparticles of perlite, perlite-TiO_2_, multi-walled carbon nanotubes (MWCNTs), and MWCNT-COOH, among others, result in the improved yields of secondary metabolites from plants such as *Hypericum perforatum* L. (Hypericaceae), *Salvia verticillata* L. (Lamiaceae), *Catharanthus roseus* (L.) G.Don (Apocynaceae), and *Salvia nemorosa* L. (Lamiaceae) [222]. Additionally, silver nanoparticles increased the production of tryptanthrin in *Isatis constricta* P.H. Davis (Brassicaceae) 1.71-fold [223]. Challenges remain, including phytotoxicity and oxidative stress associated with the use of nanoparticles as a treatment for medicinal plants. However, further research may offer a sustainable process for the common use of nanoparticles as additives to medicinal plants to increase alkaloid yields.

There has been a recent rapid development in the production of nanoparticles of alkaloids to examine their biological properties. For example, berberine (**6**) nanoparticles, prepared through the evaporative precipitation of a nanosuspension, showed enhanced antimicrobial activity against Gram-positive and Gram-negative bacteria and yeasts [224]. Encapsulation of ergot alkaloids has also provided enhanced bioactivity, by way of improved bioavailability, in ocular drug administration [225]. These two examples reveal the importance of combining nanotechnology with the drug discovery process of both known and new alkaloids.

#### 3.4.5. Repurposing and the Follow-Up of Known Alkaloids

The repurposing of the diverse scaffolds of alkaloids which have been approved for other indications [2] offers a viable approach to addressing urgent needs, or when the potential patient pool is so small that a formal discovery process through larger-scale screening could not be justified [226]. An overview of drug repurposing efforts has been compiled [227]. Any library of compounds for biological evaluation in a new test system should have a core set of alkaloid scaffolds, including all those that are established as approved drugs, as well as 50–80 additional alkaloids from sustainable sources. One example that has already shown to offer dual use is the alkaloid colchicine (**26**). Well-known as an alkaloid for the treatment of gout, it showed good effectiveness for the treatment of familial Mediterranean fever and was approved for this use in 2009 [228]. 

Due to the challenges of acquiring an adequate collection of alkaloids in a single location with appropriate bioassay capacity, alternative approaches to repurposing have been adopted. One strategy has been to target specific organisms, such as helminths [229], or a specific disease, such as diabetes [230]. The reverse process considers a set of drugs for a disease, for example immunomodulators [231], or the CNS [232], with retesting for other specifically desired indications. An in silico study of 92 alkaloids in 10 structure classes from *Papaver* species examined the structure-activity relationships through chemoinformatics and generating heatmaps for a broad range of receptors, and serves as a potential model strategy [233]. The importance of large datasets of biological options for docking is critical, as well as the need to consider the sustainability of sourcing as “hits” are accumulated. It is also important to recognize the opportunity to keep testing in silico with all the many available model targets [234].

**Figure 6:**
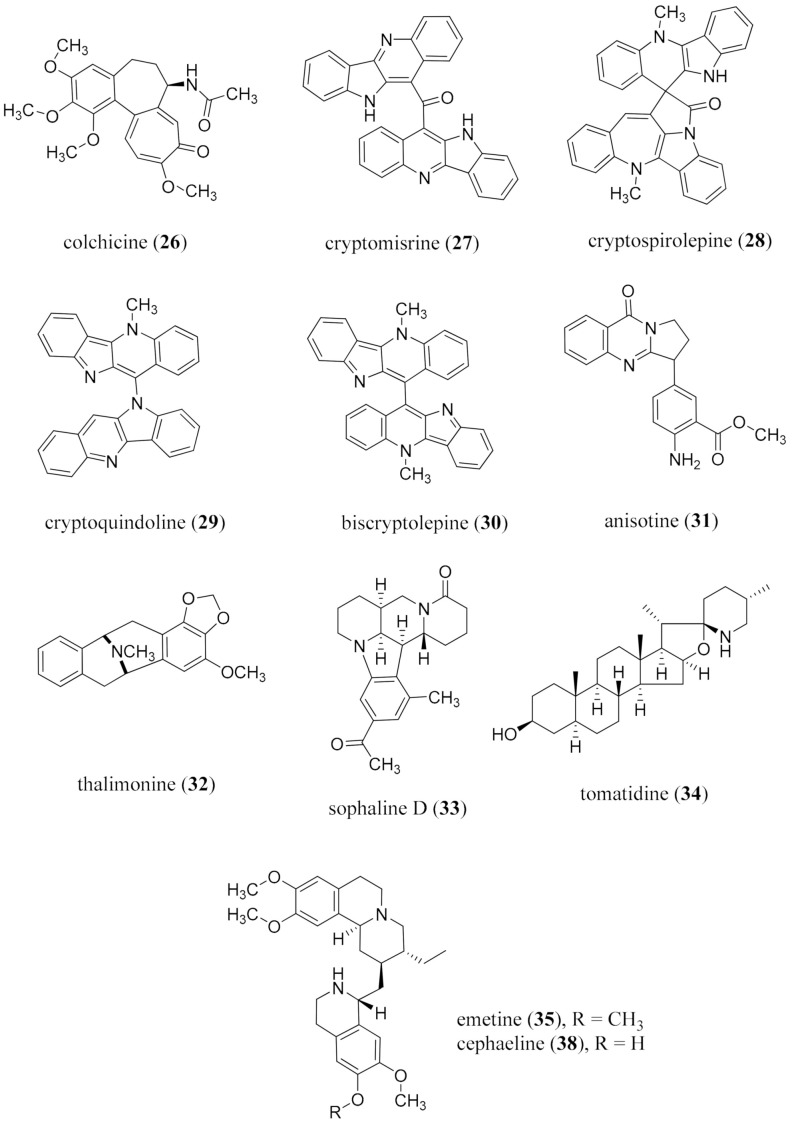
Examples of alkaloids to consider for repurposing as a drug discovery strategy.

In silico binding studies rapidly became important in discovery initiatives for the ongoing SARS-CoV-2 virus (COVID-19) pandemic, and colchicine (**26**) has been studied as a potential antiviral agent [235]. The assessment of different scaffolds for utility at various stages in the SARS-CoV-2 disease evolution is a rapidly expanding activity in drug discovery [236,237], particularly using in silico techniques [238,239,240], providing some hits for further analysis in vitro. Four of the 13 alkaloids evaluated from *Cryptolepis sanguinolenta* (Lindl.) Schltr. (Apocynaceae), namely cryptomisrine (**27**), cryptospirolepine (**28**), cryptoquindoline (**29**), and biscryptolepine (**30**), were identified as having strong binding energies with the main protease and the RNA-dependent RNA polymerase of SARS-CoV-2 [238]. Anisotine (**31**) from *Justicia adhatoda* L. (Acanthaceae) also bound strongly to the main protease [239], as did thalimonine (**32**), sophaline D (**33**), tomatidine (**34**), and emetine (**35**) in a study of 17 alkaloids (Figure 6) [241]. These approaches can also serve as a model for discovery for NTDs where critical enzymes can be identified and modeled.

**Figure 7:**
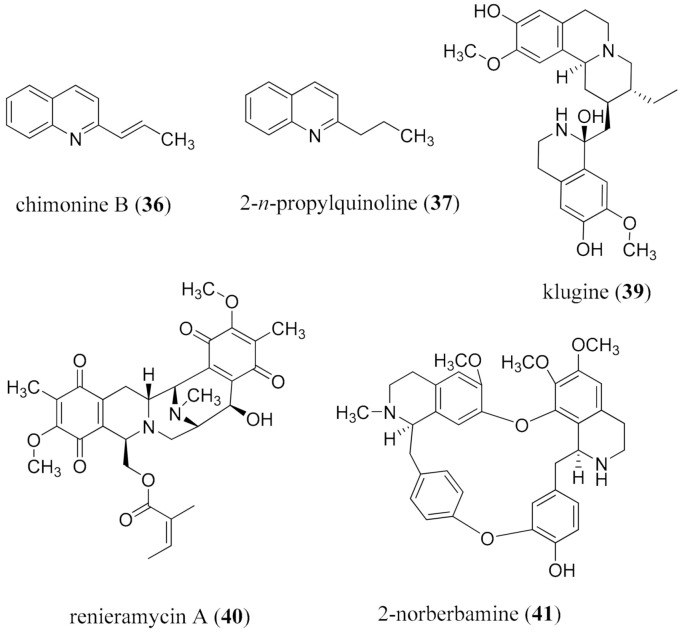
More examples of alkaloids warranting further consideration.

Another aspect of repurposing which covers resource minimization is follow-up, i.e., examining the literature, assessing the existing data, and considering whether further studies are warranted. Some examples relating to leishmaniasis and malaria will illustrate. Simple 2-alkylated quinoline alkaloids, such as chimanine B (**36**) from the stem bark and leaves of *Galipea longiflora* K. Krause (Rutaceae) and synthetic 2-propylquinoline (**37**), were effective against CL and VL in a mouse model [241,242]. This would appear to be a very promising scaffold for exploratory SAR studies, as analogues are readily synthesized. The *Psychotria* (*Cephaëlis*) alkaloids also show activity against *L. donovani* isolates [243]. Cephaeline (**38**) was active (IC_50_ 0.06 μM), but cytotoxic, whereas klugine (**39**), the 6-*O*-methyl ether derivative, was less active (IC_50_ 0.85 μM) and not cytotoxic [243]. Finally, a marine metabolite, renieramycin A (**40**) from a *Neopetrosia* species, showed an IC_50_ of 0.35 μM against *L. amazonensis* (Figure 7) [244].

The examination of 16 medicinal plants used in Thailand for malaria and available in the marketplace, in the standard *Plasmodium falciparum* assays, revealed 12 to be active, from which four groups of alkaloids (aporphines, bisbenzylisoquinolines, 5,10b-ethanophenanthridines, and tetrahydroprotoberberines) were shown for the first time to possess *selective* antimalarial activity [245]. The activity of 2-norberbamine (**41**) (SI 2400) was particularly noteworthy. Those discovery opportunities based on available medicinal plants remain to pursued. Strategies for the repurposing of alkaloids through a combination of in silico binding studies and in vitro testing, coupled with up-front considerations of sustainability and accessibility, will evolve as both a functional strategy for identifying new opportunities, and a predictive approach based on artificial intelligence applications. A quite different aspect of repurposing centers on the evolution of genomic approaches to understand all the nodes where an alkaloid is operating as a part of network pharmacology (vide infra) [246,247,248].

### 3.5. Genomics-Based Discovery

Discovering new alkaloid scaffolds from microbial sources has evolved dramatically with the availability of more complete genomes, and the bioinformatics applications supporting analysis of the data. Two disclosures have been critical in opening the opportunities for new alkaloids to be generated and tested: the structures of the biosynthetic gene clusters (BGCs), and the presence of cryptic clusters. Unlike the pathways for metabolite formation in plants, the genes encoding for the biosynthetic enzymes in microorganisms are clustered and highly organized. For polyketide synthases (PKS) and non-ribosomal peptide synthases (NRPS), they have characteristic domains for performing specific biosynthetic pathway steps. Gene clusters for certain NRPS, PKS, and NRPS/PKS hybrid alkaloids can be identified through a genome mining bioinformatics approach, as a form of dereplication and discovery [249]. 

As more gene clusters have been analyzed in silico, organisms are revealed to have the genetic capacity to produce many more metabolites in different classes than previously observed [250]. Actinobacteria were identified as a good resource [251], as well as Gram-negative bacteria in the genera, *Pseudomonas*, *Clostridium*, and *Burkholderia* [252,253], cyanobacteria [254], and marine sponge-associated bacteria [255]. Fungi (e.g., *Aspergillus* sp.) are considered an even better source of cryptic biosynthetic clusters [256]. 

These “silent” pathways can constitute the majority of the identified BGCs, and frequently represent unidentified compounds. For example, *Streptomyces coelicolor* A3(2) had six identified metabolic pathways with 16 BGCs encoding for unknown metabolites [250]. Based on the domains for substrate activation of the BGCs [257], those likely to produce alkaloids, i.e., the NRPS and NRPS/PKS clusters, are easily identified [258], and represent a significant opportunity [249,259,260]. The identification of BGCs having unrecognizable modular characteristics probably indicates new scaffolds [261,262,263] and the characterization of new alkaloid structures [264,265,266,267,268]. The profound challenge is to activate the pathways selectively, or in a heterologous host [260,269,270,271,272,273]. An excellent summary of these techniques and the range of chemical outcomes is available in [260].

At the present, a complete natural product structure cannot be predicted de novo from a new biosynthetic gene cluster; although algorithms may assist [274], heterologous expression of the BGC and conducting the isolation and structure elucidation is required. Once non-functional modules can be unambiguously identified in silico, and substrate specificities defined, levels of structure prediction for an assembled pathway product will be enhanced. 

Searching NRPS motifs will yield alkaloids. An assessment of 830 genome sequences of Actinobacteria comprised 11,422 gene clusters which were grouped into 4122 families [249]. Three taxons are considered the most important for their BGCs, the Streptomycetales, the Pseudonocardiales, and the Streptosporangiales, which account for 80% of the NRPS BGCs. Mass spectral analysis of the products from 178 strains grown in four different media revealed an average of 105 compounds/strain, represented by 2251 individual metabolites. Twenty-seven known metabolites were correlated with their BGC and verified in the samples 268 times. Hence, dereplication is necessary to avoid unwanted metabolite redundancy purification. Based on the known Actinobacteria, hundreds of thousands of metabolite scaffolds probably remain to be characterized, with closely related analogs also being produced [249]. 

### 3.6. Applications of Metagenomics 

Opportunities exist for assessing ecological environments holding clues to new drug discovery through the resident extremophiles [275] and represent a classic opportunity for metagenomics [276,277,278]. Metagenomics is a method, independent of cultivation, for the collective examination of the microbial genomes in a particular habitat [279], particularly for new metabolites [276,280,281]. It was greatly enhanced by high-throughput DNA sequencing technologies and database access to established BGC sequences. This has allowed microbial communities to be analyzed at the ecosystem level [278]. Function-based screening was a successful approach for the cyanobactin polypeptide macrolide alkaloids [282]. Achievements in identifying new, skeletally diverse alkaloids and other metabolites through the metagenomics approach, as well as the barriers to success related to the expression of genes in heterologous hosts, have been discussed [283,284]. For the future, the interlacing of machine learning with bioinformatics and synthetic biology will be crucial in the further development of metagenomics and for new alkaloid discovery.

## 4. Alkaloids and the Fourth Industrial Revolution

### 4.1. Industry 4.0 (4IR) and the Quintuple Helix

Cognizant of the limitations of “conventional technologies”, the issues associated with alkaloid drug discovery for global disease needs warrants the extensive application of integrated technologies which can offer new perspectives and approaches for more systematic and sustainable research outcomes. Seven of the twelve recognized technologies associated with the Fourth Industrial Revolution (4IR) [17] impact natural products and drug discovery, they are: artificial intelligence (AI) and AI systems, ubiquitous linked sensors, new computing technologies, 3D printing, advanced materials and nanomaterials, biotechnologies, and neurotechnologies [20]. These technologies operate in concert with the Quintuple Helix, which extends the models for driving creativity, innovation, and development in science and engineering [19]. The Triple Helix focuses on the interrelationships between academia, industry, and government for enhancing innovation in various sectors. The Quadruple Helix adds the context of a “knowledge society”, in which successive exchanges of knowledge between the scientific, engineering, and technology sectors stimulate innovation in another sector. The exchanges promote “knowledge democracy”, which, coupled with the globalization of the internet and other communications systems, allows for instantaneous and equal access to datasets of accrued knowledge irrespective of their location(s) [285]. The Quintuple Helix coalesces the ecologically sensitive component and a healthy populace with the knowledge society derived from the collaboration of academia, industry, and the government into creativity and innovation. It is fundamental to sustainable drug discovery, and due to the extensive prior data from a long history of effective use, can specifically involve alkaloids in the creative process [18,286]. This integrated approach stands as an essential aspect of the “green knowledge” solutions to human issues [287], optimizing the use of natural resources while conserving them, a component of the United Nations Sustainable Development Goals [54]. Consideration of the environmental impact and sustainability early in the drug discovery process are aspects of ecopharmacognosy [129,130]. That will inspire creativity and innovation for the potentiation of natural resources, including alkaloids, to meet global health needs, through the integration of the existing and evolving technologies in terms of cyberecoethnopharmacolomics (CEEPO) [20,288]. This level of integration, while currently not a realistic expectation for most of the world, should be considered a goal for the future.

### 4.2. Artificial Intelligence in Drug Discovery

The past fifteen years have witnessed the dramatic impact of artificial intelligence (AI) in drug discovery in the pharmaceutical industry [289,290,291,292,293,294,295], including the development of automated and complete compound design cycles as ultra-HTS systems [296]. Outside of Big Pharma, fully integrated design systems will focus on smaller, highly targeted libraries [297] which are more conducive for the assessment of characterized and semi-purified plant, marine, and fungal extracts. 

To enhance the assessment of known and new alkaloids, the speed of screening is not the key issue as the sample numbers are limited. Success will arise through generating active-site binding affinity data [298], establishing alkaloid sustainability, and the targeted assays. Advanced AI systems applications to libraries of alkaloids, selectively modified and tested on an iterative basis, in conjunction with chemically primed, 3D-printed modules introducing successive reaction steps, will become a routine practice [299,300]. 

Automated recognition of feasible synthetic processes (oxidation, reduction, hydrolysis, esterification, aromatic substitution, etc.) for a particular sustainable alkaloid structure will lead to autonomous design profiles conducted by an AI system [301]. Until a system is “trained” through machine learning, only a balance between human creativity, automation, and artificial intelligence/machine learning, will identify “leads”. Considerations of drug-off-target interactions and bioavailability assessments in early phase synthetic options will be critical for efficiency. Five “grand challenges” in the rethinking of drug design [294] were cited as: obtaining appropriate datasets, generating new hypotheses, optimizing in a multi-objective manner, reducing cycle times, and changing the research culture.

Within this discovery framework lies network pharmacology, the holistic approach of considering a single compound to have multiple nodes of action [246,247,248]. Systematic mapping through AI of the action sites at an early stage may yield indications about interactions with other drugs from a synergistic or antagonistic perspective, providing glimpses of potential toxicity issues [302], and should reduce the high levels of attrition occurring in the clinical trials stages [303]. The Universal Natural Products Database [234] comprising (in 2013) 197,201 natural products is a resource to explore natural product structure diversity, space, drug-likeness, and biological perspectives. It was docked against 332 target proteins of FDA-approved drugs, which revealed that natural products occupied a more expansive space than synthetic drugs, implying a far greater diversity of potential biological properties for future discovery, particularly as more diverse biological systems are explored [234]. Although the average number of targets for natural products was 2.66, two IMPS/PAINS compounds, the indolocarbazole alkaloid staurosporine (**42**) with 298 targets, and the widely distributed flavonoid quercetin (**43**) with 82 targets, were of particular interest [141,142,143]. The paucity of published biological reports for the vast majority (98.2%) of the natural products in the database was noted. For enhanced benefit to be derived, an important question to be asked is “What are the active site space requirements that need to be filled?”, especially as the space-filling profiles of the FDA-approved drugs are so limited. This particularly applies to the characterized NTD active sites and drives the need for further active site characterization for disease inhibition to match alkaloid scaffolds and their tailoring.

The Connectivity Map (CMap) at the Broad Institute in Cambridge, MA is an important resource for examining the impact of small molecules on gene expression. It has over 1.5 million gene expression profiles derived from ~5000 compounds and ~3000 genetic reagents, evaluated in multiple cell types, and can be used to search for compounds having a gene expression profile corresponding to a specific disease phenotype [304]. Other systems for predicting therapeutic outcomes for natural product small molecules have also been presented [248]. One outcome was identification of the quinolizidine alkaloid matrine (**44**) to act synergistically with the anti-angiogenic activity of the *enantio*-morphine alkaloid sinomenine (**45**). Interestingly, these two metabolites are important alkaloid constituents of the TCM formula Qing-Luo-Yin which is used to treat angiogenesis [305,306].

### 4.3. Machine Learning

**Figure 8:**
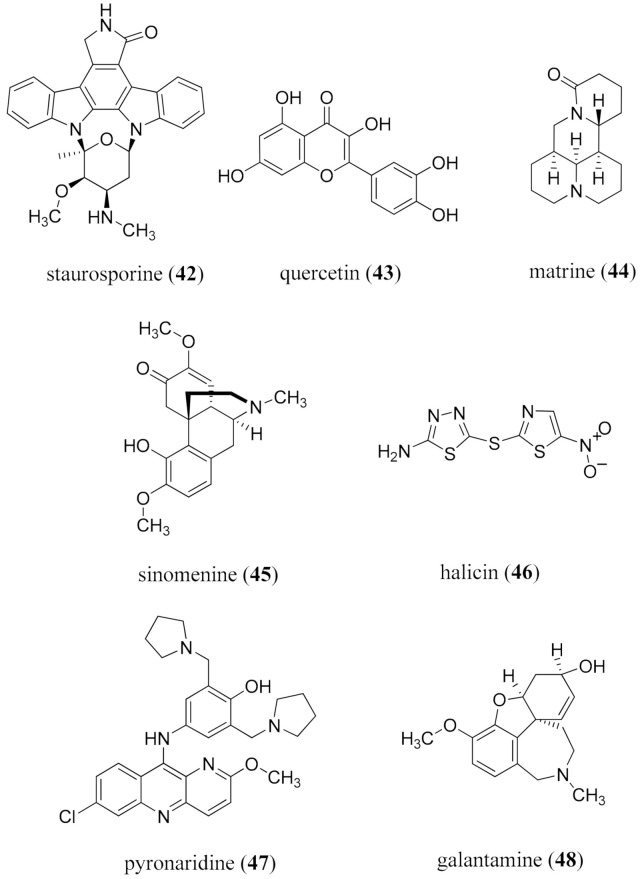
Representative bioactive alkaloids and quercetin (**43**).

Machine learning (ML) is the application of algorithms which perform explorations through pattern recognition in large data sets [307,308]. It is evolving as an essential tool in contemporary alkaloid drug discovery. The algorithms of ML may be either supervised (indicating sorting to predefined categories), or unsupervised in which clusters are created and classification occurs through application of the algorithms. The ability to adjust and “learn” during the sorting process, or processes, allows for more meaningful implications to be drawn from large datasets. Learning establishes layers of deep neural networks (DNNs) which interrelate sets of data and have become a fundamental tool in contemporary drug discovery [309,310,311] through applications to high throughput screening (HTS) and quantitative structure activity relationship (QSAR) analyses [312,313], pattern recognition [314], identifying possible drug-target interactions [315], and for detecting and monitoring drug-resistant pathogens [316]. Enhanced facility in applying algorithms to specific datasets will be an important step to improve accessibility for the non-chemoinformatics specialist. One recent successful DNN application was the identification of an interesting new scaffold for an antimicrobial in halicin (**46**) [316]. Halicin (**46**) was active against *Mycobacterium tuberculosis* and carbapenem-resistant enterobacteria in vitro, and against *Clostridioides difficile* and pan-resistant *Acinetobacter baumannii* in vivo. ML models for compounds active against Chagas’ disease using the Broad Institute set of analogs revealed five compounds which tested positive against *T. cruzi* in vivo, including the synthetic antimalarial drug pyronaridine (**47**) (Figure 8) [317].

Recent applications of machine learning have included searching for the structural parameters important for antimalarial activity [318], classifying natural and synthetic molecules in large data sets of 300 K structures and quantifying natural product likeness [319], and searching for mimetics of the expensive Alzheimer’s drug, the Amaryllidaceae alkaloid galantamine (**48**), from 3,383,942 compounds [320]. Machine learning has been used to prioritize the application of biological screens using published bioactivity data [321] and, based on spatial considerations, to classify a database of 25,523 natural products from bacterial and fungal sources [322]. An interesting outcome from the latter study was the ability to predict fungal or bacterial origin through analysis of the structure. 

“End-to-end” (E2E) machine learning in drug discovery [323] has been proposed as a standard practice across all aspects of the discovery process [324,325]. In this way the design, synthesis, identification, and the presentation and biological testing of compounds, irrespective of origin, will be enhanced [326]. Through the application of multiple algorithms, smaller corporate and academic research programs should be able to identify more compounds for assessment against rare or neglected diseases [317]. In spite of some limitations [323], the ChEMBL database may be especially useful with respect to targeting compounds and biological testing [327]. With its available expertise and dataset assets, Big Pharma could do much more to both encourage and sponsor new drug discovery efforts for NTDs and new antibiotics between small companies and academia, activities which will always be outside their discovery realm. Their role will likely occur when their clinical development capabilities would be beneficial.

A recent review summarized the global distribution of some of the databases currently operational in the natural products community, many of which are only locally accessible [328]. For effective practices in the context of the Quintuple Helix, interoperability and accessibility to create knowledge parity are key elements. One vision is to develop a natural product data portal for the input and egress of information, which would link key databases relating to taxonomy, ethnomedicine, chemistry, biology, and spectroscopy, etc. to achieve a globally accessible resource for the evolution of natural products research [329]. If achieved, this would constitute the disruptive innovation for the analysis of natural products research priorities. The sourcing and sustainability requirements are critical elements in analyzing large compound datasets as metabolites are identified for biological assessment.

The unification of machine learning and genomics discussed previously represents a powerful combination of technologies for the future diversification of natural product structures, especially alkaloids, in drug discovery, and in a targeted manner [330]. Natural products are regarded as already in nature, and thus a discovery source. New choices abound for the design and creation of new molecules from nature, an aspect of combinatorial biosynthesis. Advances in BGCs genomic information, the interweaving of large biological datasets, and the introduction of AI systems for programmed genetic manipulation processes, and for examining culturing options, in conjunction with in silico efforts to identify specific structure modifications of biological relevance, will bring focus to a fully integrated design approach for new “natural” alkaloids for testing.

Will a laboratory AI system be able to achieve those biosynthetic and biological processes in the natural product arena, as they do now for compounds derived through organic synthesis? Feedback loops for structure function relationships for in silico analysis will foster the determination of new pharmacophores which can be enhanced through machine learning. Will machine learning lead to suggestions for new functionalized alkaloid scaffolds for biosynthesis? Can that creative process be driven towards an optimized series of innovative compounds for further examination against NTDs? Such prospects are exciting indeed.

## 5. A Way Forward

In the present drug development model, the financing of drug discovery to produce a successful clinical entity in the marketplace rests with a few large pharmaceutical companies. Only 10% of potential medicinal agents survive clinical assessment, raising the estimated development costs to as high as USD 2.8 billion, depending on the therapeutic indication [331]. That level of investment is not, and cannot be, made for a single drug for the NTDs and other world diseases in need. Global pharmaceutical companies are not, and will not be, developing drugs to meet the need for drugs for the rare diseases, NTDs, malaria, or tuberculosis [55,332]. Repeating the same processes and waiting for a drug to “appear” is not a solution for a country to address the contemporary need for a new drug for the local scourge. Collaborative initiatives based on the Quintuple Helix and integration with the technologies of 4IR across borders are going to be the only way forward.

That will take putting the global patient first and will indicate that reprioritization of investment is required to create a new paradigm of drug development. Given the continuing success of natural products in drug discovery over the past 40 years [56], and the abundant natural resources in the areas of the world in greatest need of new drugs, that should be a clear natural product development priority at the international level. In this Fourth Industrial Revolution, the prime factors for a paradigm shift in natural product drug discovery are present: the unexplored biological and chemical resources of nature, the vast amounts of stored data on the chemistry and biology of natural products yet to be analyzed, the technologies for the creation of in-field biosensors, AI systems for the automated isolation of bioactive metabolites, and for the optimization of natural product structures through in silico assessment of receptor interactions at the genome level.

The transference of technologies from the developed pharmaceutical industries must become a major investment for middle-income economies where scientific (including personnel) and technological infrastructure is poised for the successful development of medicinal agents to meet local prevalent disease needs. Such initiatives will build intellectual, physical, and economic resources as new industries evolve and centers of excellence are created. Two factors are missing: intent and financing. It requires consortium commitment from governments, industry, and academia in a neutral environment, such as sponsorship through a global foundation or agency, to assemble the parties to discuss, decide on priorities, and act innovatively. Model studies with well-designed disease-based priorities, programs, and performance targets will be needed. The answer to the question of “What is the most important aspect of natural product research?” is not related to technology, or scientific infrastructure, or even information systems. It is “collaboration”. It is how natural product scientists and technologists in the many different areas embraced by the seven aspects within 4IR identified earlier can innovate at the interface of chemistry, biology, and technology to create new medicinal agents which can be safe, effective, and accessible to challenge the unmet health needs of the society. The vibrant history of alkaloids in the past 115 years indicates that, as a class of natural product, they should have a very important role to play in future global health care.

### The Need for a Third Class of Medicinal Agents

If the extraordinary costs (up to USD 2.8 billion) to develop a new drug in the present regulatory system are a barrier to drug discovery and development for diseases outside the Big Pharma paradigm, then a new framework is needed [21]. As Buckminster Fuller said “...to change something, build a new model that makes the existing model obsolete”. The creations of the internet and the smartphone are recent examples. That is the opportunity for alkaloids and the Fourth Industrial Revolution, interwoven with the development in scientific society of the Quintuple Helix. The paradigm shift required is to establish a third class of natural product drugs which cost much less to develop, are based on sustainable (local) natural sources, are a standardized preparation, are established experimentally as safe and effective, and will have gone through more limited, structured clinical trials. It is a necessary cost-effective, risk-averse step which should be promoted at the international level to address the numerous global disease issues, including multidrug resistance, malaria, tuberculosis, and the rare and neglected tropical diseases (NTDs).

## 6. Conclusions

There is much still to be learned about alkaloids and their beneficial role in health care. The marine and terrestrial biomes have enormous unexplored potential for disclosing the presence of new scaffolds. The era of 4IR and the Quintuple Helix bring together the power of large data systems, artificial intelligence, machine learning, laboratory AI systems, and knowledge exchange based on collaborative integrated and environmentally sensitive programs which can evolve for the generation of new, biologically significant molecular entities through the sustainable assessment of known alkaloids. 

A new international paradigm for natural product discovery and development is necessary to meet the medicinal agent requirements for the treatment of multidrug resistant diseases, rare and neglected tropical diseases, and for malaria and tuberculosis. The natural, physical, and technological resources are present or under rapid development. Fiscal commitment and international consortia with intent are needed to transform the existing paradigm and envision an approach which optimizes the rapid developments of 4IR with the innovations created within the Quintuple Helix for these diseases. A third class of drugs will foster these developments and inspire both investment and innovation. Through the application of contemporary technologies applied through human creativity, new, repurposed, and redesigned alkaloids will make an important contribution to these and other drug discovery efforts. A new and exciting era for alkaloid research is already underway.
